# Risk factors for discontinuation of exclusive breast feeding within 1month: a retrospective cohort study in Japan

**DOI:** 10.1186/s13006-022-00449-w

**Published:** 2022-03-05

**Authors:** Shun Yasuda, Toma Fukuda, Naoya Toba, Norihito Kamo, Karin Imaizumi, Midori Yokochi, Tomoko Okawara, Seiko Takano, Hideko Yoshida, Nobuko Kobayashi, Shingo Kudo, Kyohei Miyazaki, Mamiko Hosoya, Kenichi Sato, Kei Takano, Aya Kanno, Tsuyoshi Murata, Hyo Kyozuka, Akiko Yamaguchi, Fumihiro Ito, Shinichiro Oda, Nobuo Momoi, Mitsuaki Hosoya, Keiya Fujimori

**Affiliations:** 1 Departments of Obstetrics and Gynecology, Iwase General Hospital, Sukugawa City, Fukushima Prefecture Japan; 2 Department of Obstetrics and Gynecology, Fukushima Medical University, Fukushima City, Fukushima Prefecture Japan; 3 Department of Pediatrics, Iwase General Hospital, Sukugawa City, Fukushima Prefecture Japan; 4 Department of Pediatrics, Fukushima Medical University, Fukushima City, Fukushima Prefecture Japan

**Keywords:** Breastfeeding, Smoking, Japan, Postpartum period

## Abstract

**Background:**

While breastfeeding provides benefits for infants and the mother, many women either do not breastfeed or terminate breastfeeding earlier than recommended. The aim of this analysis was to identify factors associated with early discontinuation of breastfeeding in Japanese women.

**Methods:**

This study used data from medical records of women delivering a singleton live birth between March 2017 and August 2019 in Iwase General Hospital, Fukushima Prefecture, Japan to assess cessation of breastfeeding by the 1-month postpartum appointment. Demographic (age at birth, and employment status), medical (parity, and physical and mental condition of the mother; and infant medical factors, such as sex, Apgar score, and jaundice, among other), and family factors (husband/partner, family members living at the same house, among others) in 734 women who had initiated breastfeeding during their delivery hospital stay were examined, and multiple logistic regression was used to determine significant predictors of early cessation of exclusive breastfeeding.

**Results:**

Bivariate analysis revealed that women who were primipara, unmarried, exposed to secondhand smoke, and employed; those who smoked before pregnancy; and those who had asthma were more likely to discontinue exclusive breastfeeding than other women. Infant factors associated with discontinuation were lower birthweight, earlier gestational age, neonatal intensive care unit admission, treatment for jaundice, or lower weight gain. Multivariable analysis revealed that primiparity, passive smoking before pregnancy, maternal employment, and neonatal jaundice therapy were associated with discontinuation of breastfeeding.

**Conclusions:**

In particular, women whose partners smoked before pregnancy may need to be targeted for additional support for breastfeeding.

## Background

Breastfeeding provide optimal nutrition for babies. Infants who are breastfed have lower rates of infection, better cognition, and a lower risk of obesity than those who are not. However, the extent to which this relation is causal, especially in developed countries, is not completely clear. Breastfeeding also has important benefits for maternal health, including reduced risk of depression [1] and reduced cardiovascular and metabolic risk later in life [2]. These benefits are most strongly seen in women who breastfeed and in infants who are breastfed for a long period of time [3, 4], it follows that early cessation reduces these improvements. In Japan, > 95% pregnant women attempt to breastfeed their infants, but around 42–49% could exclusively breastfeed during the first month postpartum [5]. Breastfeeding is strongly encouraged, and clinicians work together to establish breastfeeding before a woman leaves the hospital after delivery. However, many women discontinue breastfeeding shortly after delivery, even within the first month, well before supplementation is optimal.

Many women experience difficulties breastfeeding, including physical symptoms (cracked nipples, mastitis, among others) and logistical barriers such as returning to work [6, 7]. In addition, breastfeeding is a dynamic interaction, and women who believe that they are not providing sufficient quantities of milk or that their infant is not gaining a sufficient amount of weight are more likely to supplement or discontinue breastfeeding altogether [6, 8–10].

The goal of this analysis was to identify physical, medical, socioeconomics and cultural factors associated with early discontinuation of breastfeeding, with a particular focus on factors relevant to women in Japan, where breastfeeding is highly socially accepted, but exclusive breastfeeding for the recommended time is less common compared to that in other countries [11–13]. Breastfeeding practices are not universal, and previous studies on predictors of breastfeeding in Japan have found mixed associations between breastfeeding and age and education factors that are strongly associated with breastfeeding in other countries [5]. Previous studies have indicated that living with the baby’s father promotes breastfeeding, while living with a grandparent does not [14]. The information from this study data will assist with the development of clinical practice and other programs that may mitigate the risk of early discontinuation of breastfeeding.

## Methods

### Study setting and population

This study was conducted at Iwase General Hospital, the only general public hospital serving the Fukushima Prefecture in Japan. The hospital has a small neonatal intensive care unit (3 beds) that can treat infants born at ≥ 34 weeks of gestation and performs 50 deliveries per month. In Japan, 99% women deliver in a hospital or clinic, and the average length of hospital stay associated with delivery is 5 days. The hospital follows the World Health Organization’s guidelines for establishing breastfeeding, including facilitating skin-to-skin contact, allowing rooming-in, and not providing breastmilk substitutes [15].

### Study design

This retrospective cohort used data from the electronic medical records from a single hospital. Medical records of women delivering a singleton live birth between March 2017 and August 2019 were included in this analysis; a total of 1350 women met this criterion. Women were excluded if they delivered preterm (< 37 weeks of gestation, *n* = 77), delivered an infant with birthweight < 2500 g or < -1.5 SD of the mean for gestational age (*n* = 148) and remained in the neonatal intensive care unit (NICU) at time of mother’s discharge (*n* = 104), or had any serious maternal complications (HELLP—hemolysis, elevated liver enzymes, low platelet count—syndrome, *n* = 2 and severe postpartum hemorrhage, *n* = 2). A total of 1104 women with no obvious contraindication to breastfeeding at the time of birth, 734 (66.4%) women had established breastfeeding by the time they were discharged from the hospital (5 days postpartum), without of a doula or midwife help. All women returned to the hospital for the 1-month infant/maternal visit.

### Outcome

The outcome was discontinuation of exclusive breastfeeding by the 1-month postpartum visit, defined as complete cessation of breastfeeding or supplementation with infant formula. The outcome was assessed using a standard questionnaire and during a clinician interview about the child’s nutrition; the responses were (1) complete breastfeeding, includes expressed breast milk; (2) supplemental infant formula, given at any time from discharge (5-days postpartum) to the 1-month checkup, even if only in little quantity, in addition to breast milk; and (3) infant formula at the time of the 1-month checkup, the infant was being exclusively fed formula.

### Predictors

Demographic, medical, behavioral, and family factors for both the mother and infant were examined as possible predictors of breastfeeding discontinuation. All factors were extracted from the women’s medical records. The demographic factors examined were age at birth, marital status, and employment status (any job at time of birth). The maternal medical factors examined were parity, underlying diseases (physical condition, such as thyroid disease or diabetes, or mental condition, such as anxiety or mood disorder), allergies, pre-pregnancy weight/body mass index (BMI), type of delivery (cesarean or vaginal), use of infertility treatment (assisted reproductive technology), hemorrhage, and mastitis. The infant medical factors were birthweight, gestational age, sex, Apgar score, umbilical artery pH, admission of infant to NICU after day 1, jaundice, and weight gain per day. The behavioral factors examined were pre-pregnancy smoking, alcohol consumption (any/none), use of dietary supplements, and preference for exclusive breastfeeding (asked during the first visit to the hospital, in the prenatal interview, not just before delivery, and are a summary of their interest in breastfeeding). No women admitted to active or passive smoking during pregnancy. The examined family factors were presence of husband/partner, family members living at the same house, housing (single family/multiple dwelling), and returned to parents’ house to give birth. Adequacy of breastfeeding was calculated according to the bibliography. The median number of breastfeeding/day was 11 ± 3 (with a range frequency between 6 and 18 times per day); women feeding for more and fewer times than the range were classified into the “probably adequate” and “possibly inadequate” breastfeeding groups, respectively [16].

### Analysis

Women who were exclusively breastfeeding at the 1-month checkup were compared to those who were supplementing breastfeeding with formula or those exclusively using formula. Chi-square tests (Fisher’s exact test when cell size was < 5) were used to analyze categorical/dichotomous variables, and t-tests were used for continuous variables. Pairwise correlations between the variables were calculated, and the correlation matrix was presented graphically using the ggcorrplot in R (version 3.5.3). Variables that showed a statistically significant difference (*p* < 0.05) in bivariate analysis were included in a logistic model to identify the strongest predictors. All analyses were conducted using SPSS v. 26.

This study was approved by the Ethics Committee of Iwase General Hospital (#191,102).

## Results

Maternal and infant predictors are shown in Table 1. The study population is described in Table 2. The total smoking rate before pregnancy was 17.3%, and the smoking rate among women aged < 30 years was statistically higher than that among women aged ≥ 30 years (22.5% vs. 13.2%, *p* < 0.01). Women were less likely to continue breastfeeding if they were unmarried (7.2% unmarried among those who supplemented with formula vs. 2.9% unmarried among those who continued exclusive breastfeeding, *p* = 0.01), smoked before pregnancy (26.8% vs. 13.5%, *p* < 0.01), had a partner who smoked (34.4% vs. 23.1%, p < 0.01), were employed (70% vs. 61.8%, *p* = 0.04), or had asthma (13.2% vs. 6.7%, p < 0.01). Infant factors associated with discontinuation of breastfeeding were lower birthweight (mean birthweight 3017 g in infants who were supplemented vs. 3105 g in infants exclusively breastfed), earlier gestational age (39 weeks and 4 days vs. 39 weeks and 5 days, p < 0.01), NICU admission (12.9% vs. 7.6%, p < 0.01), neonatal jaundice treatment (12.0% vs. 5.5%, *p* < 0.01), and lower weight gain (42.4 vs. 45.7 g/day, *p* < 0.01).

**Table 1:** Maternal and infant factors in Japanese women

Predictor (*n* = 734)	*n* (%) or mean (SD)
Demographics	
Primiparity	349 (47,5%)
Age in years	
< 20	24 (3.3%)
20–30	300 (40.9%)
30–40	391 (53.3%)
≥ 40	19 (2.6%)
Unmarried	32 (4.3%)
Employed	261 (35.6%)
Period to return to work after delivery	
≤ 2 months	12 (1.6%)
3–6 months	28 (3.8%)
7–12 months	140 (19.1%)
≥ 13 months	25 (3.4%)
Health behavior	
Active smoking before pregnancy	127 (17.3%)
Passive smoking before pregnancy	175 (23.8%)
Alcohol use before pregnancy	135 (18.4%)
Dietary supplement use	212 (28.9%)
Medical History	
Pre-pregnancy weight (kg)	53.8 (9.4)
Pre-pregnancy BMI (*n* = 685)	21.6 (3.5)
Weight gain during pregnancy (*n* = 679)	9.9 (4.3)
Food allergy	69 (9.4%)
Drug allergy	48 (6.5%)
Bronchial asthma	60 (8.2%)
Underlying maternal disease	95 (12.9%)
Mastitis	47 (6.4%)
Infertility treatment	48 (6.5%)
Breastfeeding Behaviour	
Preference for EBF if possible (*n* = 429)	425 (99.1%)
Number of times of breastfed/day at one month (*n* = 650)	10.6 (3.5)
Family and housing	
Returned to parents’ house to give birth	183 (24.9%)
Post-discharge living environment	
Nuclear family	52 (7.1%)
Living with own family	88 (12.0%)
Living with husband/partner’s family	27 (3.7%)
Detached house	128 (17.4%)
Infant factors	
Vaginal delivery	614 (83.7%)
Birthweight	3090.7 (294.3)
Gestational age	39w4d (1w0d)
Male infant	363(49.5%)
5-min Apgar score	8.9 (0.4)
NICU admission	67 (9.1%)
Neonatal jaundice treatment	54 (7.4%)
Umbilical pH	7.32 (0.53)
Weight gain at 1-month check-up (*n* = 659)	44.7 (10.8)

Abbreviations: *BMI*: body mass index, *EBF* exclusive breastfeeding

**Table 2:** Maternal and infant factors associated with discontinuation of exclusive breastfeeding in Japanese women

Predictor (n with data)	Exclusive breastfeeding [n (%) or mean (SD)]	Non-Exclusive Breastfeeding [n (%) or mean (SD)]	*P*-value
Demographics			
Primiparity (*n* = 734)	229 (43.6%)	120 (57.4%)	< 0.01
Age in years (*n* = 734)	30.0 (4.7)	29.6 (6.0)	0.31
< 20	11(2.1%)	13 (6.2%)	
20–30	219 (41.7%)	81 (38.8%)	
30–40	280 (53.3%)	111(53.1%)	
≥ 40	15 (2.9%)	4 (1.9%)	
Unmarried (*n* = 734)	15 (2.9%)	15 (7.2%)	0.01
Employed (*n* = 728)	322 (61.8%)	145 (70.0%)	0.04
Period to return to work after delivery (*n* = 205)			0.11
≤ 2 months	10 (6.8%)	4 (6.9%)	
3–6 months	17 (11.6%)	10 (17.2%)	
7–12 months	102 (69.4%)	43 (74.1%)	
≥ 13 months	18 (12.2%)	1 (1.7%)	
Health behavior			
Active smoking before pregnancy (*n* = 734)	71 (13.5%)	56 (26.8%)	< 0.01
Number of cigarettes smoked (*n* = 734)			< 0.01
< 1 cigarettes/day	454 (86.5%)	153(73.2%)	
1–20 cigarettes/day	69 (13.1%)	56 (26.8%)	
≥ 21 cigarettes/day	2 (0.4%)	0 (0%)	
Passive smoking before pregnancy (*n* = 654)	103 (23.1%)	72 (34.4%)	< 0.01
Number of cigarettes smoked by household members (*n* = 611)			< 0.01
< 1 cigarettes/day	342 (82.8%)	134(69.2%)	
1–20 cigarettes/day	64 (51.4%)	60(30.3%)	
≥ 21 cigarettes/day	7 (1.7%)	1 (0.5%)	
Alcohol use before pregnancy (*n* = 732)	90 (17.2%)	45 (21.5%)	0.17
Dietary supplement use (*n* = 732)	142 (27.1%)	70 (33.5%)	0.09
Medical History			
Pre-pregnancy weight (kg) (*n* = 734)	53.8 (9.3)	53.7 (9.2)	0.98
Pre-pregnancy BMI (n = 685)	21.5 (3.5)	21.7 (3.7)	0.53
Weight gain during pregnancy (*n* = 679)	9.8 (4.3)	10.2 (4.2)	0.29
Food allergy (*n* = 734)	47 (9.0%)	22 (10.5%)	0.49
Drug allergy (*n* = 734)	38 (7.2%)	10 (4.8%)	0.32
Bronchial asthma (*n* = 701)	33 (6.7%)	27 (13.2%)	< 0.01
Underlying maternal disease (*n* = 734)	32 (6.1%)	16 (7.7%)	0.51
Mastitis (*n* = 734)	39 (7.5%)	8 (3.8%)	0.09
Infertility treatment (*n* = 734)	32 (6.1%)	16 (7.7%)	0.51
Breastfeeding Behaviour			
Preference for EBF if possible (*n* = 429)	304 (99.4%)	121 (98.4%)	0.33
Number of times of breastfed/day at one month (*n* = 650)	11.0 (3.6)	10.2 (3.0)	0.01
Family and housing			
Returned to parents’ house to give birth (*n* = 690)	135 (27.7%)	48 (23.6%)	0.27
Post-discharge living environment (*n* = 167)			0.91
Nuclear family	33 (29.5%)	19 (34.5%)	
Living with own family	59 (52.7%)	29 (52.7%)	
Living with husband/partner’s family	20 (17.9%)	7 (12.7%)	
Detached house (*n* = 158)	91 (84.3%)	37 (74.0%)	0.13
Infant factors			
Vaginal delivery (*n* = 734)	444 (84.6%)	170 (81.3%)	0.29
Birthweight (*n* = 734)	3105 (294)	3017 (285)	< 0.01
Gestational age (*n* = 734)	39w5d (1w0d)	39w4d (1w0d)	< 0.01
Male infant (*n* = 734)	248 (47.2%)	115 (55.0%)	0.06
5-min Apgar score (*n* = 734)	8.9 (0.4)	8.9 (0.3)	0.81
NICU admission (*n* = 734)	40 (7.6%)	27 (12.9%)	0.11
Neonatal jaundice treatment (*n* = 734)	29 (5.5%)	25 (12.0%)	< 0.01
Umbilical pH (*n* = 734)	7.32 (0.55)	7.31 (0.48)	0.11
Weight gain at 1-month check-up (*n* = 659)	45.7 (10.3)	42.4 (11.5)	< 0.01

Abbreviations: *BMI* body mass index, *EBF* exclusive breastfeeding

**Fig. 1:**
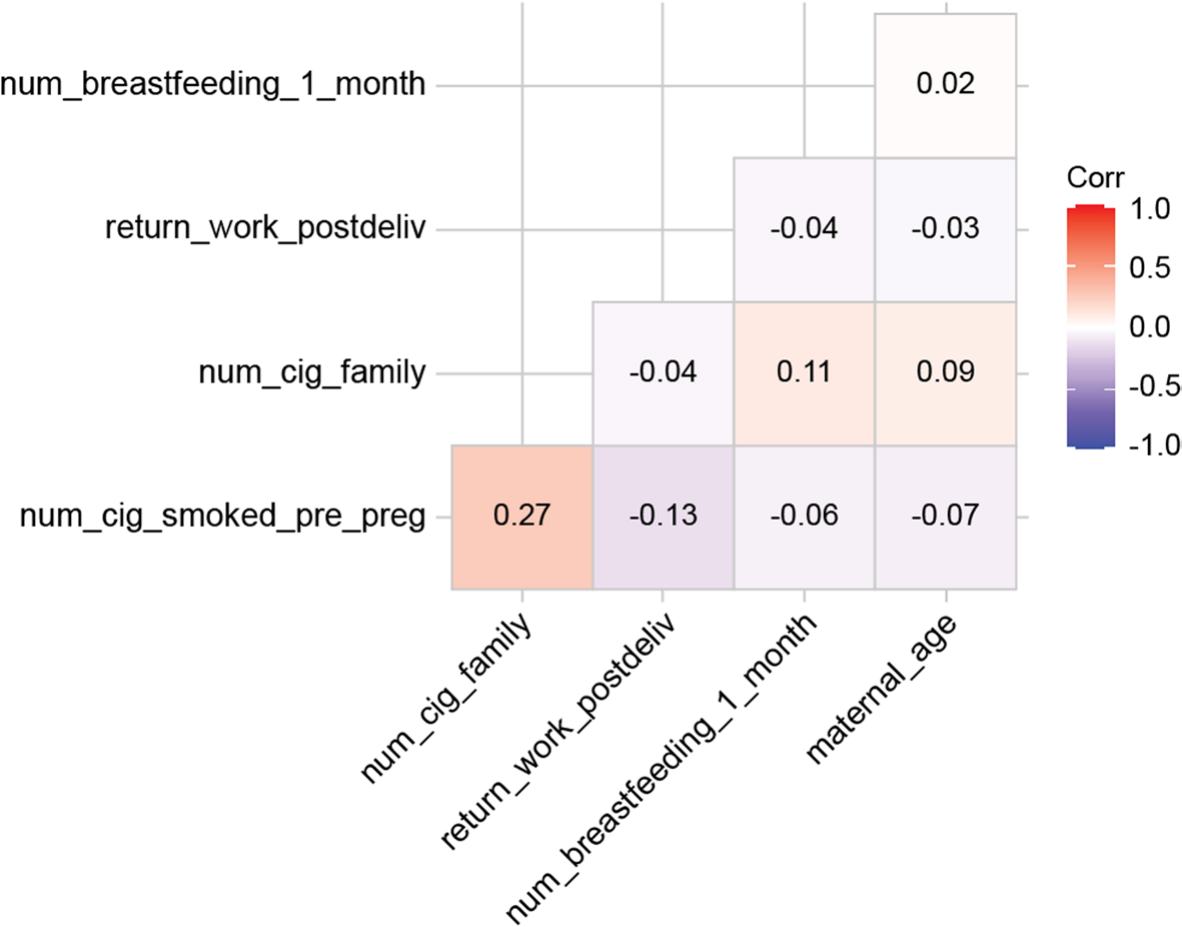
Correlation matrix for study variables. A heat map of the pairwise correlations between the variables were calculated, and was presented using the ggcorrplot. Correlations of |r|> 0.10 have *p* < 0.05. Abbreviations: EBF: cig: cigarette; exclusive breastfeeding; num: number; preg: pregnancy; postdeliv: postdelivery

Some covariates were significantly correlated (Fig. 1). The number of cigarettes smoked by the participant and the family was correlated (*r* = 0.27). Weak correlations were seen between adequacy of breastfeeding at 1 month and number of cigarettes smoked before pregnancy (*r* = -0.06), number of cigarettes smoked by household members (*r* = 0.11), maternal age (*r* = 0.02), and how quickly employees planned to return to work after delivery (*r* = -0.04).

Multivariable analysis (Table 3) revealed that primiparity (adjusted odds ratio [aOR], 1.59; 95% confidence interval [CI], 1.13,2.22), passive smoking before pregnancy (aOR, 1.52; 95% CI, 1.04,2.23), maternal employment (aOR, 1.54; 95% CI, 1.07,2.22), and neonatal jaundice treatment (aOR, 1.84; 95% CI, 1.02,3.30) were associated with discontinuation of breastfeeding during the 1-month of postpartum. The aOR for active smoking before pregnancy was not statistically significant (aOR, 1.50, 95% CI, 0.98,2.31).

**Table 3:** Multivariable predictors of discontinuation of exclusive breastfeeding among Japanese women

	aOR	95% CI
Primiparity	1.59	1.13,2.25
Employed	1.54	1.07,2.22
Unmarried	1.69	0.78,3.67
Active smoking before pregnancy	1.50	0.98,2.31
Passive smoking before pregnancy	1.52	1.04,2.23
Neonatal jaundice treatment	1.84	1.02,3.30

Abbreviations: *aOR* adjusted odds ratio, *CI* confidence interval

## Discussion

Although breastfeeding was well established when mothers were discharged from hospital after giving birth, primiparity, maternal employment, passive smoking before pregnancy, and infant jaundice requiring phototherapy were associated with an increased risk of discontinuation of breastfeeding. The associations between breastfeeding and primiparity and maternal employment have been well established. Breastfeeding and parental employment are associated in the breastfeeding duration, but not in the breastfeeding initiation. Mothers who return to work after a year of childbirth left breastfeeding duration before mothers who left employment before childbirth. In addition, fathers with a flex time system work after childbirth, breastfeeding initiation increases and the duration of breastfeeding is prolonged, suggesting that work flex time could be a strategy to promote breastfeeding in Japan [17].

Passive smoking due to active smokers in the household was a predictor of breastfeeding discontinuation. Exposure to secondhand smoke has been associated with reduced likelihood of breastfeeding [18], breastfeeding cessation [19, 20] and with reduced duration of breastfeeding [21, 22]. Passive smoking has been associated with changes in breastmilk lipids [23], which might alter infant growth and contribute to discontinuation. Reverse causality is also a possibility; if the mother has ceased breastfeeding, family members may be less concerned about exposing her to smoke and women may worry that chemicals may be passed to the baby through breastmilk. However, encouraging breastfeeding is particularly important in infants exposed to passive smoking, as it may mitigate some negative effects on growth [24].

In this study, mothers who smoked before pregnancy show a controversial tendency. Based on the type of analysis, as categorical/dichotomous variables or bivariate analysis, the significance change. Even there was a small risk, with a large number of patience the tendency would be stronger. Although maternal pre-pregnancy smoking was not a predictor of the outcome in this study, number of cigarettes smoked was highly correlated between participants and family members. This suggests that smoking cessation efforts may need to target the whole family. In this study, although all pregnant women who smoked before pregnancy were strictly discouraged from smoking and no women admitted to either active or passive smoking during pregnancy, it was also possible that some women continued smoking during pregnancy, as the self-reports were not confirmed via biomarker tests. It appears that women who smoke has lower motivation to breastfeed, rather than a physiological effect of smoking on their milk supply, than women who does not smoke [25]. To support pregnant women in breastfeeding, a programme of accompaniment and support for all family members to reduce or cease smoking must be carried out. Further research is needed on the best way to accomplish this. Smoking cessation through pediatric practices rather than primary or gynecologic care practices shows promise [26]. Several evidence-based programs to improve cessation during pregnancy and prevent relapse have been shown to be effective [27], although not all have been tested in a Japanese population and relapse rates are quite high [28]. A combination of behavioral and incentive strategies may be the most effective [29].

The epidemiology of smoking in the region is particularly concerning. Smoking before pregnancy was more common in younger women (aged < 30 years) than in older women (aged ≥ 30 years), which is concerning for regional public health as it implies that the prevalence of smoking may be increasing among women of reproductive age. Fukushima Prefecture has one of the highest smoking rates in Japan. According to the Cancer Registry and Statistics of the Cancer Information Service of the National Cancer Center, Japan, as of 2016, 10.7% of women aged ≥ 20 years were smokers in Fukushima Prefecture, ranked as the eighth worst prefecture in Japan. The pre-pregnancy smoking rate among women who deliver in our hospital is even higher at 23%.

Neonatal jaundice treatment was associated with an increased likelihood of discontinuation of breastfeeding. A previous Canadian study found no differences in discontinuation between infants hospitalized for hyperbilirubinemia and those who were not [30]. Similarly, an Italian study failed to find jaundice to be a risk factor [31]. However, as “breastfeeding jaundice” is a common finding shortly after birth and may be alarming to parents, it seems reasonable that its presence might contribute to discontinuation of breastfeeding.

Several factors that are generally considered to be associated with breastfeeding cessation, in this analysis shown no association. After multivariate analysis, the association between discontinuation of breastfeeding and lower infant weight gain was too small to be considered clinically relevant. Previous studies have identified pre-pregnancy BMI and pregnancy weight gain as important predictors (occasionally interacting with smoking) [32], which was not the case in the current analysis. In this study, differences in birthweight for gestational age (a factor shown to be associated with discontinuation in other studies [33]) by breastfeeding status were identified; however, these differences were not considered clinically significant and, consequently, were not included in further analysis.

The strengths of this study include the consideration of medical, individual, and family factors as well as the extended follow-up. Furthermore, as none of the women reported smoking during pregnancy, the study isolated the effect of pre-pregnancy active and passive smoking. The limitations of the current study include the lack of information on maternal mental health during pregnancy or postpartum (at the time of the study, depression was not usually included in the medical record) or difficulties in breastfeeding beyond mastitis. In addition, no information was available on attitudes and intentions regarding breastfeeding [34], pacifier use (not recommended by the hospital) [35], or previous breastfeeding among multiparous women. Moreover, other studies have explored various factors that were not assessed in this study. For instance, formula supplementation in the hospital has been shown to be associated with an increased likelihood of cessation [36]. Intention to breastfeed and length of intended time are also strongly associated with each other [37, 38]. In addition, perceived insufficient milk supply, breast problems, and tiredness were associated with discontinuation of breastfeeding before 3 months of delivery in a cohort of women who intended to continue breastfeeding [36]. Lack of confidence in breastfeeding, belief that the baby prefers formula, young maternal age, and lower education were associated with cessation of breastfeeding in the first 2 weeks after delivery in a cohort of lower-income women in Connecticut (USA) [38].

## Conclusions

Exposure to smoking before pregnancy, either as actively or passively smoker, is a risk factor for discontinuation of breastfeeding. Other factors could affect the adequacy of breastfeeding, such as the number of cigarettes smoked, maternal age, and the plan of return to work.

This epidemiological study shows that preconception education is required to encourage women and families members to quit smoking before they begin planning pregnancy. Even the underlying mechanism are beyond the scope of this study, is clear that further studies on smoking cessation in household members and mothers as a component of preconception care are also planned.

## Data Availability

The datasets generated and analyzed in the current study are not publicly available due to patient confidentiality concerns but are available from the corresponding author on reasonable request.

## Abbreviations

BMI: Body mass index
EBF: Exclusive breastfeeding
NICU: Neonatal intensive care unit
aOR: Adjusted odds ratio
CI: Confidence interval
